# The Interplay Between Gut Microbiota, Adipose Tissue, and Migraine: A Narrative Review

**DOI:** 10.3390/nu17020337

**Published:** 2025-01-18

**Authors:** Valentina Biagioli, Federica Mela, Paola Ferraro, Gianmichele Villano, Alessandro Orsini, Maria Cristina Diana, Pasquale Striano, Andrea Santangelo

**Affiliations:** 1 Department of Neurosciences, Rehabilitation, Ophthalmology, Genetics, Maternal and Child Health, University of Genoa, 16126 Genoa, Italy; 2 Pediatric Neurology and Muscular Diseases Unit, IRCCS Istituto Giannina Gaslini, 16147 Genoa, Italy; 3 Pediatric Neurology, Pediatric Department, AOUP Santa Chiara Hospital, 56100 Pisa, Italy

**Keywords:** microbiota–gut–brain axis, adipose tissue, diet, migraine, neuroinflammation

## Abstract

Background: Migraine, a prevalent neurovascular disorder, affects millions globally and is associated with significant morbidity. Emerging evidence suggests a crucial role of the gut microbiota and adipose tissue in the modulation of migraine pathophysiology, particularly through mechanisms involving neuroinflammation and metabolic regulation. Material and Methods: A narrative review of the literature from 2000 to 2024 was conducted using the PubMed database. Studies addressing the relationships between microbiota, adipose tissue, and migraine—including dietary interventions and their impact—were analyzed. Results: The findings highlight a bidirectional gut–brain axis, with gut microbiota influencing neuroinflammation via metabolites such as short-chain fatty acids (SCFAs). Obesity exacerbates migraine severity through chronic inflammation and the dysregulation of adipocytokines like leptin and adiponectin. Dietary patterns, such as low glycemic index diets and Mediterranean diets, and the use of prebiotics, probiotics, and postbiotics show potential in migraine management. Conclusions: This review underscores the need for integrative approaches targeting the microbiota–gut–brain axis and adipose tissue in migraine therapy. Future studies should explore longitudinal effects and personalized interventions to optimize outcomes.

## 1. Introduction

Migraine is a complex and recurrent disorder that significantly affects the global population, with its prevalence rising from 2.80% in 2008 to 14% in 2022 [1]. Chronic migraine, defined as experiencing headaches for at least 15 days per over a three-month period, has a prevalence of 0.8% to 1.8% in children aged 12 to 17 years [2]. The prevalence increases from childhood to adolescence, and differs by gender, affecting boys and girls equally in childhood but disproportionately impacting females after puberty [3].

From a clinical perspective, migraine is characterized by episodic pulsatile headaches, often accompanied by nausea, vomiting, photophobia, and phonophobia, which can significantly reduce quality of life (QoL) [4]. Current pharmacological approaches target acute and preventive strategies. Acute medications include triptans (e.g., sumatriptan and rizatriptan), which act by constricting blood vessels and inhibiting pain pathways, as well as nonsteroidal anti-inflammatory drugs (NSAIDs). However, these treatments focus on alleviating symptoms rather than addressing the underlying causes of migraine, and are often associated with limited efficacy and the recurrence of attacks [5]. Therefore, it is urgent to develop disease management strategies, capable of preventing when possible and attenuating the factors that precipitate and perpetuate the clinical picture.

**Figure 1:**
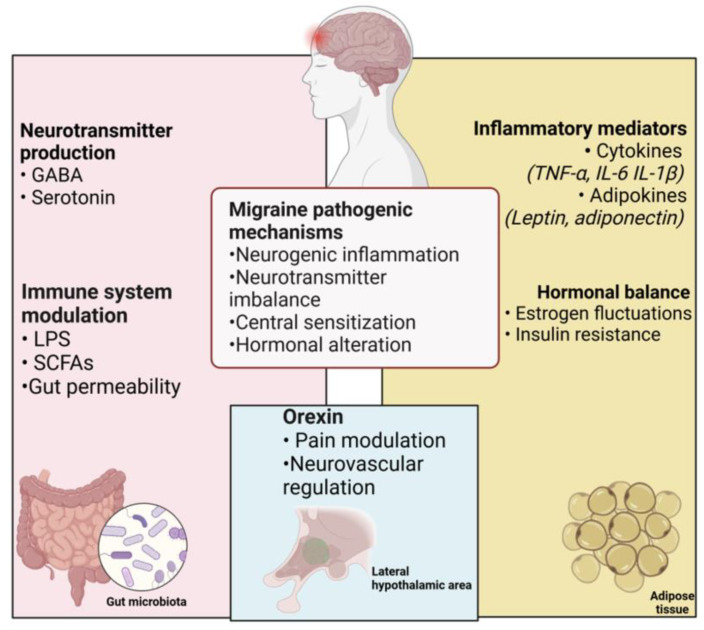
Gut microbiota and adipose tissue can modulate neuroinflammation and immune system response acring on several migraine pathophysiology mechanisms.

While non-modifiable risk factors such as genetic predisposition, age, and sex play a significant role, modifiable factors—including lifestyle, diet, physical activity, and obesity—offer substantial opportunities for intervention [6]. Emerging evidence suggests that diet and nutrition are pivotal in influencing the pathophysiology of migraine. In fact, nutritional factors could modulate several metabolic pathways [7], impacting neurotransmitter levels (e.g., GABA and serotonin) and glucose, lipid, and protein metabolism, as well as microbiota composition. Numerous studies have demonstrated that glutamate can exert an excitatory activity on nociceptive neurons through the trigeminovascular pathway, contrary to GABA. In light of this evidence, good microbial biodiversity is fundamental for maintaining the factors precipitating and triggering migraines [8]. Therefore, these factors collectively influence neuroinflammation, a key mechanism in migraine development (Figure 1). Therefore, foods such as alcoholic beverages, chocolate, caffeine, and dairy products have been identified as potential “triggers” for migraines, although the evidence remains inconclusive due to conflicting results [9,10].

In light of new scientific discoveries regarding the role of the microbiota in human health, targeted dietary interventions have been proposed as promising approaches to migraine [11]. Moreover, Chen J. et al., have demonstrated how, in migraine patients, the biodiversity and intestinal microbial richness are reduced, favoring the Clostridium species instead (e.g., *Cl. asparagiforme*, *Cl. clostridioforme*, *Cl. ramosum*) [12]. This review explores the multifaceted relationship between diet, microbiota, and migraine, aiming to elucidate potential pathways for novel, integrative therapeutic strategies that address the root causes of this debilitating condition.

## 2. Materials and Methods

A comprehensive search was performed using PubMed, Scopus, and EBSCO databases to identify relevant literature published between 2000 and 2024. Search terms included “microbiota”, “migraine”, “diet”, “neuroinflammation”, and related phrases. Boolean operators (AND, OR) were used to refine search results, and the search was supplemented with the backward citation tracking of key articles. We included original research articles, systematic reviews and meta-analyses published in English or Italian, addressing the microbiota–gut–brain axis, dietary factors, or obesity in relation to migraine, while case reports and letters were excluded. Both human and animal studies were reviewed to provide a comprehensive understanding of the topic.

A total of 201 abstracts were initially retrieved (105 from PubMed, 51 from EBSCO, and 45 from Scopus); after removing duplicates, 150 records were screened. After removing the papers not assessing migraine, abstracts, narrative reviews, and meeting abstracts, 69 papers were included in the review.

Titles and abstracts were screened independently by two reviewers to identify potentially relevant articles. Full-text screening was subsequently performed for eligibility. Discrepancies between reviewers were resolved through discussion or consultation with a third reviewer.

The results were organized into thematic areas, including the microbiota–gut–brain axis (25 articles), dietary influences on migraine (16 papers), and the role of obesity (11 articles). Both descriptive and comparative analyses were conducted to highlight patterns, gaps, and future research directions.

## 3. Results in the Literature

### 3.1. Microbiota–Gut–Brain Axis and Role of Inflammation in Migraine

Humans are not only made of human cells; each hosts a total bacterial mass of 200 g [9]. The set of these microorganisms that cohabit with the host organism symbiotically and commensally, and can interact with each other through complex mechanisms of cross-feeding, constitutes a single functional unit defined “Holobiont” [13].

The environmental niches (intestine, vagina, mucous membranes, skin, etc.) that the host organism offers to microorganisms must guarantee their survival through pH, temperature, and nutrients [14]. These microorganisms (including viruses, archaea, bacteria and fungi) can produce metabolites, modulate neurotransmitters, and influence the metabolism of vitamins and drugs [15]. In particular, the intestinal microbiota can establish a complex axis defined as the microbiota–gut–brain axis (MGBA), which allows for communication between the gut and CNS either unidirectionally, via metabolites that cross the blood–brain barrier (BBB), or bidirectionally though the vagus nerve [16]. Additionally, the Hypothalamic–Pituitary–Adrenal axis (HPA), by modeling cortisol levels, can alter intestinal permeability [17], and eventually even neurons themselves can intervene in regulating this complex axis [18]. Moreover, in the gut, immune cells and inflammatory cytokines produced, such as interleukins (IL-1, IL-6), tumor necrosis factor (TNF-α), and C-reactive protein (CRP), seem to be involved in migraine pain, worsening the severity of attacks [19]. Understanding the complex bidirectional interaction between our gut, the microorganisms that inhabit it, and the host’s immune system remains a topic of great scientific interest.

Short-chain fatty acids (SCFAs), including acetate, butyrate, and propionate, are crucial for maintaining intestinal balance (eubiosis) [20], and can reach the CNS by crossing the portal circulation. At the CNS level, they perform numerous functions, such as promoting neuronal proliferation and differentiation, exerting neuroprotective effects, promoting the production of brain-derived neurotrophic factor (BDNF), reducing pro-inflammatory cytokines (suppressing TNF alpha) and the endotoxic activity of lipopolysaccharide (LPS) [21]. Interestingly, preclinical studies showed that hypernociception induced by pro-inflammatory conditions is reduced in germ-free mice compared to controls. Moreover, Bai J et al. examined the associations between the gut microbiome and migraines in children aged 7–18 from the American Gut Project. They showed that children with migraines had lower diversity and higher abundances of inflammatory-related bacteria, such as *Eggerthella, Sutterella*, and *Eubacterium*. In contrast, children without migraines had higher abundances in the phylum *Firmicutes, Christensenellaceae*, and *Ruminococcaceae*. It was shown that *Eggerthella* has been linked to clinically significant bacteremia and underlying GI diseases, suggesting a high level of pathogenicity; furthermore, elevated levels of *Eggerthella* are linked to mental disorders, such as depression, bipolar disorder, and schizophrenia; on the other hand, it was shown that the protective mechanism of *Ruminococcaceae* may be attributed to its prebiotic functions, such as butyrate production and starch fermentation abilities in the GI tract, promoting the growth of other beneficial bacteria. *Ruminococcaceae* has also been found to be negatively correlated with inflammatory markers [22]. In a study by Danielle G. Souza et al., germ-free mice exhibited no local, remote, or systemic inflammatory response following intestinal ischemia–reperfusion injury, unlike conventional mice, which showed significant edema, neutrophil influx, hemorrhage, and elevated TNF-α levels. Germ-free mice produced higher levels of the anti-inflammatory cytokine IL-10, both post injury and after LPS administration, with minimal TNF-α production. These findings underscore the microbiota’s critical role in driving inflammatory responses and regulating immune signaling [23].

### 3.2. Migraine and Gastrointestinal Disorders: Is There a Link?

Over 70% of migraine patients exhibit one or more gastrointestinal disorders (GIDs) [24], whereas patients suffering from inflammatory bowel diseases, celiac disease, irritable bowel syndrome, cyclic vomiting syndrome, functional dyspepsia, gastroparesis, and peptic ulcers appear to have a greater prevalence of migraines [25]. Notably, the occurrence of migraines increases progressively with the number of GIDs [26]. Several theories attempt to explain the correlation between these two clinical presentations. It is believed that the autonomic nervous system plays a significant role in the association between migraines and GI, given the similarities in their symptom profiles, which include nausea, vomiting, dyspepsia, and gastroparesis [27]. Pro-inflammatory cytokines such as IL-1β, IL-6, IL-8, and TNF-α are implicated in both migraines and GIDs [28], contributing to visceral pain and systemic inflammation. Neuropeptides, particularly CGRP (calcitonin gene-related peptide), play a central role in migraine pathophysiology and are also expressed in the gastrointestinal tract [29]. The β-CGRP isoform, predominant in the enteric neurons [30], is linked to GI symptoms such as gastroparesis, constipation, and diarrhea, which often co-occur with migraines. Notably, elevated CGRP levels exacerbate both migraines and GI symptoms [31]. Additionally, gut microbiota alterations further influence CGRP signaling, as seen in experimental studies where germ-free mice, particularly females, showed increased CGRP production [32]. The release of CGRP from parasympathetic perivascular and trigeminal fibers during a migraine, along with changes in intestinal microbiota and gut permeability in response to stressors, can lead to the release of pro-inflammatory mediators, which in turn affect nociceptive responses in the trigeminal pathway contributing to migraine development [33]. Serotonin also bridges the gut and brain, acting as a pain modulator and regulator of gastric emptying. Reduced activity of serotonin 5-HT1B/1D receptors activates the trigeminovascular system, contributing to migraine attacks. The dysregulation of serotonin pathways is further associated with delayed gastric emptying and other GI dysfunctions [34].

### 3.3. Obesity–Migraine Linkage

The World Health Organization (WHO) defines overweight and obesity as the abnormal or excessive accumulation of weight, with a BMI ≥ 25 kg/m^2^ indicating overweight and ≥30 kg/m^2^ indicating obesity [35]. These conditions are rising globally in both adults and children, representing a true global pandemic influenced by malnutrition in the forms of excess, deficiency, and the so-called “hidden hunger” which primarily affects micronutrients [36].

The link between obesity and migraine remains unclear, although both conditions share common mechanisms such as chronic systemic inflammation, dysregulation of appetite, and dysbiosis [37]. Diet and lifestyle have shown promising effects on both conditions. Hershey et al. conducted a study on 913 pediatric headache patients, demonstrating a significant association between obesity and headache frequency. A reduction in BMI correlated with fewer headache episodes [38]. Similarly, Kinik et al. found obesity to be more prevalent among children with migraine compared to the general population, with obese children experiencing more frequent attacks [39]. However, obesity and migraine are two chronic diseases, whose connection has yet to be clarified.

## 4. Discussion

### Neurotransmitters and Postbiotics: The Silent Messengers of the Microbiota

**Figure 2:**
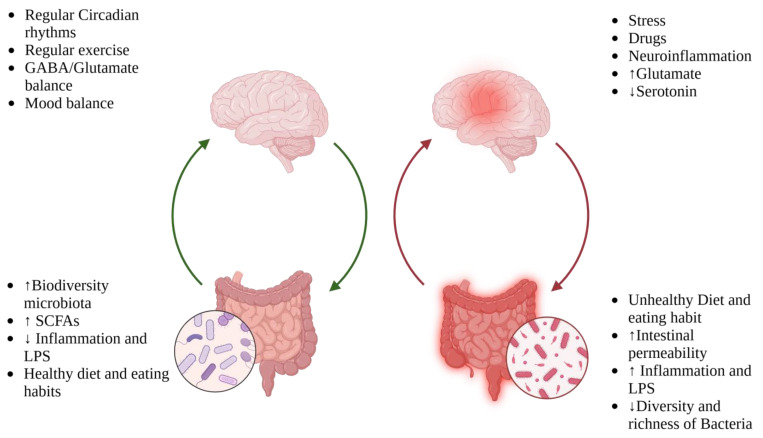
Bidirectional communication in the MGBA and how different factors can influence the pathophysiological mechanisms underlying gut–microbial balance and migraine.

The hypothalamus regulates food intake and appetite, but migraine patients often exhibit hypothalamic dysfunction, including altered sleep–wake cycles, mood changes, and hunger–satiety dysregulation (Figure 2). Neurotransmitters like serotonin, adipokines, and orexin, many metabolized by the gut microbiota, play dual roles in appetite regulation and migraine pathogenesis. Elevated plasma CGRP levels, observed in obese women and mouse models of obesity, link obesity-related inflammation with migraine exacerbation [40].

Serotonin is a neurotransmitter, 90% of which is produced by enterochromaffin cells (EECs) and only 5–10% by serotonergic neurons in the brain [37]. Its precursor, tryptophan, derived from foods such as dairy, eggs, and fish [41], is metabolized by gut microbiota into 5-hydroxytryptophan, from which EECs produce 5-hydroxytryptamine (5-HT) [42]. Serotonin locally promotes peristalsis, while centrally regulates mood, nociception, and the sense of hunger–satiety [43]. Studies on mouse models show that blocking the 5-HT2C receptor is associated with the development of obesity and increased food intake [44]. Furthermore, in subjects with migraine, there are low levels of serotonin in the interictal states, with a consequent increase in food intake [45].

GABA, an inhibitory neurotransmitter, is studied extensively in patients with epilepsy; omic studies have identified the presence of GABA-producing bacterial strains such as the *Bifidobacterium adolescentis* strain. *B.adolescentis* is the most abundant bifidobacterium in the large intestine [46]. This species, thanks to its vast enzymatic repertoire, can degrade sugars, starting from dietary prebiotic molecules such as resistant starch carbohydrates. A low presence of *B.adolescentis* is associated with a state of intestinal dysbiosis and the development of allergies, diabetes mellitus type 1, type 2, and IBD [47]. Moreover, several studies suggest that dysbiosis and low GABA levels occur following severe migraine attacks and that it plays a role in suppressing migraine attacks [48].

Adipokines are cytokines produced by adipose tissue. Adiponectin, an anti-inflammatory molecule participating in glucose homeostasis, is inversely correlated with diabetes, metabolic syndrome, cardiovascular diseases, and chronic low-grade inflammation [49]. Furthermore, the concentration of high molecular weight adiponectin is statistically higher (*p* < 0.005) in patients suffering from chronic migraine (6.1 ± 2.8 μg/mL), compared to subjects who suffer from it episodically (4.2 ± 1.7 μg/mL) and healthy subjects (3.9 ± 1.5 μg/mL) [50]. Leptin, which regulates glucose homeostasis and inflammation, is elevated in obesity [51]. Leptin receptors are widespread in the hypothalamus, cortex, and brain endothelium, and their pathway involves NFkB, e-NOS, and AMPK, the same pathways implicated in migraine [52]. Domínguez C et al. measured levels of leptin, adiponectin, and other inflammatory markers (IL-6, IL-10, TNF alfa, high sensitivity PCR-hsPCR) related to migraine pathophysiology in a group of migraine patients and healthy controls, highlighting that leptin and adiponectin serum levels were increased in migraine patients and were significantly higher in chronic compared to episodic migraine patients (*p* < 0.001). Furthermore, they found a positive correlation between leptin levels and the following inflammatory biomarkers: IL6 (r = 0.498; *p* < 0.001), TNF-a (r = 0.389; *p* < 0.001), and hs-CRP (r = 0.422; *p* < 0.001) [53]. These findings link adipocytokines to migraine pathophysiology, supported by Rubino et al., who also observed elevated adiponectin and resistin levels in chronic migraine patients after adjusting for BMI, sex, and age [54].

Orexin, a hypothalamic neuropeptide regulating appetite and the sleep–wake cycle, shows altered levels in migraineurs’ CSF. Interestingly, preclinical studies associated orexin with increased food-seeking behavior under low glucose conditions, suggesting a role in migraine triggers [55].

Lipopolysaccharide (LPS), a pro-inflammatory molecule from Gram-negative bacteria, enters systemic circulation during dysbiosis, causing neuroinflammation [56]. A recent Italian study on a cohort of pediatric migraine patients showed higher levels of plasma occludin and IgA, associated with increased intestinal permeability, and an increase in plasma LPS, indicating low-grade intestinal inflammation compared to healthy controls [57].

## 5. Diet: What We Know and Future Directions

### 5.1. Diet Triggers

Dietary factors may influence migraines through biochemical mechanisms, including the modulation of neuropeptides, neuroreceptors, and cerebral glucose metabolism, as well as by triggering inflammation, nitric oxide (NO) release, vasodilation, and vasoconstriction. In a study by Taheri et al., chocolate triggered migraines in 22% of children, likely due to flavanoids increasing NO levels and phenylalanine stimulating norepinephrine release, leading to vasoconstriction [58]. Moreover, caffeine was linked to 28% of headaches, acting either as a trigger or via withdrawal.

In another study, the gradual cessation of cola consumption in 36 individuals resulted in complete cessation of all headaches in 33 cases [59]. On the other hand, symptoms of coffee withdrawal may occur typically 12–24 h after cessation, and approximately 47% of individuals experience a migraine as a result of caffeine abstinence. This occurs because caffeine has a chemical structure like adenosine, a neuromodulator involved in the regulation of migraine, so it acts as a nonselective antagonist of adenosine receptors. The chronic blocking of adenosine receptors by caffeine appears to increase the risk of migraine, because it seems to result in an increased sensitivity to adenosine, which is evident when caffeine is withdrawn [60].

Fasting can induce migraines, particularly around 16 hours into a fast, through hypoglycemia, stress hormone release, and altered serotonin levels. Gum chewing was also identified as a trigger that may increase the risk of migraine attacks. A prospective study conducted by N. Watemberg et al. displayed how discontinuing lead to complete symptom resolution in 63% (19/30) of children with chronic headaches, and partial improvement in 23% (7/30), whereas the reintroduction of the habit led to relapse in 20/26 children [61].

Managing migraines through trigger identification, fiber-rich diets, low glycemic index diets, and supplementation with vitamin D, omega-3, and probiotics can promote gut health and reduce symptoms. Weight management also benefits migraineurs with obesity [62].

### 5.2. Elimination Diet

The elimination diet involves identifying and removing foods that trigger migraines, often based on patient’s self-reported food–symptom correlations [63]. This mechanism, however, and therefore the cause–effect search, could be misleading and not lead to a resolution of the problem, as the relationship is not as linear; often, it may not be the food itself that causes the problem but other possible biases, such as stress, a lack of adequate rest, and dehydration [64]. Foods are considered triggers if they consistently provoke migraines in >50% of cases within a day of consumption. However, confounding factors like stress, dehydration, and poor sleep may misattribute the cause. When supervised by nutrition specialists, elimination diets can help identify triggers, but may risk malnutrition if entire food groups are excluded without proper reintroduction protocols [65].

### 5.3. Dash and Mediterranean Diet

Recent studies show how Dietary Approaches to Stop Hypertension (DASH), have proven to be a useful nutritional regime in adults to reduce the frequency, severity, and duration of migraine attacks. Initially designed to treat patients with hypertension, it focuses on reducing sodium, saturated fats, and simple sugars (mainly sweets), promoting the consumption of whole grains, fruit, and vegetables [66]. Another dietary regime considered the gold standard not only in the prevention of diseases but also in their treatment is the Mediterranean diet, rich in fruit, vegetables, legumes, and seeds and low in saturated fats, simple sugars, and refined carbohydrates. The Mediterranean diet, thanks to the PREDIMED study, has attracted strong interest in the scientific community precisely because of its protective effects from cardiovascular risk, even in subjects predisposed to the development of cardiovascular diseases (hypertension, type 2 diabetes, and smokers) [67]. This nutritional regime appears to bring very similar results to the DASH in migraine patients.

### 5.4. Low Glycemic Index Diet

A low glycemic index diet is a diet that is based on the inclusion of foods that have a low glycemic index, so less than <50 [68]. Foods with a high GI such as white bread, rice, and sugary desserts lead to a high postprandial glucose peak compared to foods with a low GI; among the low GI foods, we have those included in the Mediterranean diet, such as legumes, seeds, non-starchy vegetables, and whole carbohydrates. For patients who suffer from migraine, a profile of greater glycemic stability appears to bring greater benefits, also improving insulin sensitivity [69]. In a randomized controlled study, in a cohort of patients with migraine, the effect of LGID and pharmacological therapy (topiramate and flunarizine) was evaluated for a duration of 90 days, and both interventions had a beneficial effect on the frequency of monthly migraine attacks and the VAS scale.

### 5.5. Functional Food and Administrations of Supplements: Prebiotics, Probiotics and Postbiotics

The FAO defines functional foods as foods that, in addition to containing macronutrients, can, thanks to specific nutrients, be beneficial to health [70]. Thus, functional foods have a positive impact on the individual’s health that goes beyond simple basic nutrition. Similarly, thanks to the new definitions provided in 2016 by the International Scientific Association for Probiotics and Prebiotics (ISAPP), prebiotics have defined as a substrate that is used selectively by the host microorganisms, thus providing proven beneficial effects on health [71]; while probiotics, defined by FAO and ISAPP in 2001, are live microorganisms that, if administered in adequate quantities, confers benefits to the host organism. Among the prebiotics useful for human health, we find Human Milk Oligosaccharides (HMOs), galactooligosaccharides (GOS), fructooligosaccharides (FOS), inulin, and resistant starches [72]. These are useful in co-administration with probiotics whose bacterial strains will be able, through fermentation mechanisms, to provide metabolites useful both for the host and cross-feeding mechanisms. For example, *Bifidobacterium longum infantis*, *Bifidobacterium breve*, and *Bifidobacterium bifidum* can metabolize HMOs, thus promoting correct microbial colonization [73]. In patients with migraine, probiotic supplementation has been shown to be successful in reducing migraine attacks [74]. A possible mechanism of action is the promotion by bacteria of SCFAs, thus promoting the integrity of the intestinal epithelial barrier and reducing the inflammatory cascade through the transduction of the NF-kB signal. This mechanism of action is also favored by *Akkermansia Muciniphila*; scientific evidence on murine models has shown how *A. Muciniphila* can interact with the Toll-like receptor at the apical level of enterocytes, and this bond favors the transduction for genes that express tight junctions, such as occludin and claudin. This mechanism promotes microbial compartmentalization, preventing the possible migration of LPS at the systemic level, in turn promoting a pro-inflammatory activation [75]. Furthermore, *A. Muciniphila* is able to increase the expression of serotonin transporters. In an open-label trial, on a cohort of 40 patients with migraine, they received supplementation with probiotics, minerals, and vitamins, for 12 weeks; at the end of the study, 80% of the patients showed a reduction in migraine attacks and an improvement in the quality of life [61]. Moreover, Martami F. et al. studied a sample of 40 patients with episodic migraine and 39 chronic migraine patients that were provided a mixture of probiotics for 10 weeks or a placebo; the results were a significant reduction in migraine attacks and their severity in the group that received the supplementation compared to the placebo [76]. However, to date, there is a lack of longitudinal studies with larger sample sizes; furthermore, the limitations of the studies present in the literature involve often not defining which probiotic strain is used, and the lack of data investigating the nutritional aspect, which we have observed to be a fundamental driver for microbial eubiosis and, consequently, the disease outcome.

## 6. Conclusions

This review has highlighted the multidirectional relationship underlying the microbiota, neuroinflammation, migraine, and obesity. These factors strongly influence the patient’s QoL, often consequently altering their mental health. Furthermore, they represent widespread pathologies that impact the direct and indirect costs borne by society at the healthcare level [77]. For these reasons, there is an urgency and a need for a paradigm shift. The risk in medicine, which is focused only on the symptom, is losing sight of the wholeness that characterizes the human body, composed not only of cells and tissues but also of a microbial consortium capable of modulating numerous aspects of our health. Further research and clinical studies are necessary to understand what the appropriate dietary model is for migraine patients and what the possible frontiers may be in the field of prebiotic, probiotic, and postbiotic integration that can be included in the therapeutic path.

## References

[1] Grangeon L., Lange K.S., Waliszewska-Prosół M., Onan D., Marschollek K., Wiels W., Mikulenka P., Farham F., Gollion C., Ducros A. (2023). Genetics of migraine: Where are we now?. J. Headache Pain.

[2] Youssef P.E., Mack K.J. (2020). Episodic and chronic migraine in children. Dev. Med. Child. Neurol..

[3] Loh N.R., Whitehouse W.P., Howells R. (2022). What is new in migraine management in children and young people?. Arch. Dis. Child..

[4] Abu-Arafeh I., Howells R. (2024). Management of migraine in children and adolescents. Handb. Clin. Neurol..

[5] Patniyot I., Qubty W. (2020). Short-term Treatment of Migraine in Children and Adolescents. JAMA Pediatr..

[6] Seng E.K., Martin P.R., Houle T.T. (2022). Lifestyle factors and migraine. Lancet Neurol..

[7] Gazerani P. (2023). Diet and migraine: What is proven?. Curr. Opin. Neurol..

[8] Santangelo A., Corsello A., Spolidoro G.C.I., Trovato C.M., Agostoni C., Orsini A., Milani G.P., Peroni D.G. (2023). The Influence of Ketogenic Diet on Gut Microbiota: Potential Benefits, Risks and Indications. Nutrients.

[9] Moskatel L.S., Zhang N. (2022). Migraine and Diet: Updates in Understanding. Curr. Neurol. Neurosci. Rep..

[10] Correnti E., Cascio S.L., Cernigliaro F., Rossi R., D’agnano D., Grasso G., Pellegrino A., Lauria B., Santangelo A., Santangelo G. (2023). Idiopathic Non-Dental Facial Pain Syndromes in Italian Children: A Clinical Case Series. Life.

[11] Chen J., Wang Q., Wang A., Lin Z. (2020). Structural and Functional Characterization of the Gut Microbiota in Elderly Women with Migraine. Front. Cell. Infect. Microbiol..

[12] Chen-Liaw A., Aggarwala V., Mogno I., Haifer C., Li Z., Eggers J., Helmus D., Hart A., Wehkamp J., Lamousé-Smith E.S.N. (2025). Gut microbiota bacterial strain richness is species specific and limits therapeutic engraftment. Nature.

[13] Biagioli V., Volpedo G., Riva A., Mainardi P., Striano P. (2024). From Birth to Weaning: A Window of Opportunity for Microbiota. Nutrients.

[14] The Integrative HMP (iHMP) Research Network Consortium (2019). The Integrative Human Microbiome Project. Nature.

[15] Chen Y., Zhou J., Wang L. (2021). Role and Mechanism of Gut Microbiota in Human Disease. Front. Cell. Infect. Microbiol..

[16] Yue Q., Cai M., Xiao B., Zhan Q., Zeng C. (2022). The Microbiota-Gut-Brain Axis and Epilepsy. Cell. Mol. Neurobiol..

[17] Frankiensztajn L.M., Elliott E., Koren O. (2020). The microbiota and the hypothalamus-pituitary-adrenocortical (HPA) axis, implications for anxiety and stress disorders. Curr. Opin. Neurobiol..

[18] Biagioli V., Sortino V., Falsaperla R. (2024). Role of Human Milk Microbiota in Infant Neurodevelopment: Mechanisms and Clinical Implications. Children.

[19] Vuralli D., Akgor M.C., Dagidir H.G., Onat P., Yalinay M., Sezerman U., Bolay H. (2024). Microbiota alterations are related to migraine food triggers and inflammatory markers in chronic migraine patients with medication overuse headache. J. Headache Pain.

[20] Morrison D.J., Preston T. (2016). Formation of short chain fatty acids by the gut microbiota and their impact on human metabolism. Gut Microbes.

[21] Fan S., Guo W., Xiao D., Guan M., Liao T., Peng S., Feng A., Wang Z., Yin H., Li M. (2023). Microbiota-gut-brain axis drives overeating disorders. Cell Metab..

[22] Bai J., Shen N., Liu Y. (2023). Associations between the Gut Microbiome and Migraines in Children Aged 7-18 Years: An Analysis of the American Gut Project Cohort. Pain Manag. Nurs..

[23] Souza D.G., Vieira A.T., Soares A.C., Pinho V., Nicoli J.R., Vieira L.Q., Teixeira M.M., At V. (2004). The essential role of the intestinal microbiota in facilitating acute inflammatory responses. J. Immunol..

[24] Park J.W., Cho Y.-S., Lee S.Y., Kim E.-S., Cho H., Shin H.E., Suh G.I., Choi M.-G. (2013). Concomitant functional gastrointestinal symptoms influence psychological status in Korean migraine patients. Gut Liver.

[25] Kim J., Lee S., Rhew K. (2022). Association between Gastrointestinal Diseases and Migraine. Int. J. Environ. Res. Public Health.

[26] Aurora S.K., Kori S.H., Barrodale P., McDonald S.A., Haseley D. (2006). Gastric stasis in migraine: More than just a paroxysmal abnormality during a migraine attack. Headache.

[27] Cámara-Lemarroy C.R., Rodriguez-Gutierrez R., Monreal-Robles R., Marfil-Rivera A. (2016). Gastrointestinal disorders associated with migraine: A comprehensive review. World J. Gastroenterol..

[28] Ramachandran R. (2018). Neurogenic inflammation and its role in migraine. Semin. Immunopathol..

[29] De Bortoli N., Tolone S., Frazzoni M., Martinucci I., Sgherri G., Albano E., Ceccarelli L., Stasi C., Bellini M., Savarino V. (2018). Gastroesophageal reflux disease, functional dyspepsia and irritable bowel syndrome: Common overlapping gastrointestinal disorders. Ann. Gastroenterol..

[30] Russo A.F., Hay D.L. (2023). CGRP physiology, pharmacology, and therapeutic targets: Migraine and beyond. Physiol. Rev..

[31] Ailani J., Kaiser E.A., Mathew P.G., McAllister P., Russo A.F., Vélez C., Ramajo A.P., Abdrabboh A., Xu C., Rasmussen S. (2022). Role of Calcitonin Gene-Related Peptide on the Gastrointestinal Symptoms of Migraine-Clinical Considerations: A Narrative Review. Neurology.

[32] He Q., Wang W., Xiong Y., Tao C., Ma L., Ma J., You C. (2023). A causal effects of gut microbiota in the development of migraine. J. Headache Pain.

[33] Hindiyeh N., Aurora S.K. (2015). What the Gut Can Teach Us About Migraine. Curr. Pain Headache Rep..

[34] Coleman N.S., Marciani L., Blackshaw E., Wright J., Parker M., Yano T., Yamazaki S., Chan P.Q., Wilde K., Gowland P.A. (2003). Effect of a novel 5-HT3 receptor agonist MKC-733 on upper gastrointestinal motility in humans. Aliment. Pharmacol. Ther..

[35] Di Cesare M., Sorić M., Bovet P., Miranda J.J., Bhutta Z., Stevens G.A., Laxmaiah A., Kengne A.-P., Bentham J. (2019). The epidemiological burden of obesity in childhood: A worldwide epidemic requiring urgent action. BMC Med..

[36] Prentice A.M. (2023). The Triple Burden of Malnutrition in the Era of Globalization. Nestle Nutr. Inst. Workshop Ser..

[37] Jahromi S.R., Martami F., Morad Soltani K., Togha M. (2023). Migraine and obesity: What is the real direction of their association?. Expert. Rev. Neurother..

[38] Hershey A.D., Powers S.W., Nelson T.D., Kabbouche M.A., Winner P., Yonker M., Linder S.L., Bicknese A., Sowel M.K., McClintock W. (2009). Obesity in the pediatric headache population: A multicenter study. Headache.

[39] Kinik S.T., Alehan F., Erol I., Kanra A.R. (2010). Obesity and paediatric migraine. Cephalalgia.

[40] O’Mahony S.M., Clarke G., Borre Y.E., Dinan T.G., Cryan J.F. (2015). Serotonin, tryptophan metabolism and the brain-gut-microbiome axis. Behav. Brain Res..

[41] Chaput J.-P. (2014). Sleep patterns, diet quality and energy balance. Physiol. Behav..

[42] Agus A., Planchais J., Sokol H. (2018). Gut Microbiota Regulation of Tryptophan Metabolism in Health and Disease. Cell Host Microbe.

[43] Yano J.M., Yu K., Donaldson G.P., Shastri G.G., Ann P., Ma L., Nagler C.R., Ismagilov R.F., Mazmanian S.K., Hsiao E.Y. (2015). Indigenous bacteria from the gut microbiota regulate host serotonin biosynthesis. Cell.

[44] van Galen K.A., Ter Horst K.W., Serlie M.J. (2021). Serotonin, food intake, and obesity. Obes. Rev..

[45] Hamel E. (2007). Serotonin and migraine: Biology and clinical implications. Cephalalgia.

[46] Leser T., Baker A. (2023). *Bifidobacterium adolescentis*—A beneficial microbe. Benef. Microbes.

[47] Fan L., Qi Y., Qu S., Chen X., Li A., Hendi M., Xu C., Wang L., Hou T., Si J. (2021). *B. adolescentis* ameliorates chronic colitis by regulating Treg/Th2 response and gut microbiota remodeling. Gut Microbes.

[48] Vieira D., Naffah-Mazacoratti M., Zukerman E., Soares C.S., Alonso E., Faulhaber M., Cavalheiro E., Peres M. (2006). Cerebrospinal fluid GABA levels in chronic migraine with and without depression. Brain Res..

[49] Straub L.G., Scherer P.E. (2019). Metabolic Messengers: Adiponectin. Nat. Metab..

[50] Peterlin B.L., Sacco S., Bernecker C., Scher A.I. (2016). Adipokines and Migraine: A Systematic Review. Headache.

[51] Obradovic M., Sudar-Milovanovic E., Soskic S., Essack M., Arya S., Stewart A.J., Gojobori T., Isenovic E.R. (2021). Leptin and Obesity: Role and Clinical Implication. Front. Endocrinol..

[52] Abbasi M., Noori-Zadeh A., Seidkhani-Nahal A., Kaffashian M., Bakhtiyari S., Panahi S. (2019). Leptin, adiponectin, and resistin blood adipokine levels in migraineurs: Systematic reviews and meta-analyses. Cephalalgia.

[53] Domínguez C., Vieites-Prado A., Pérez-Mato M., Sobrino T., Rodríguez-Osorio X., López A., Campos F., Martínez F., Castillo J., Leira R. (2018). Role of adipocytokines in the pathophysiology of migraine: A cross-sectional study. Cephalalgia.

[54] Rubino E., Vacca A., Govone F., Gai A., Boschi S., Zucca M., De Martino P., Gentile S., Pinessi L., Rainero I. (2017). Investigating the role of adipokines in chronic migraine. Cephalalgia.

[55] Strother L.C., Srikiatkhachorn A., Supronsinchai W. (2018). Targeted Orexin and Hypothalamic Neuropeptides for Migraine. Neurother. J. Am. Soc. Exp. Neurother..

[56] Tuomi K., Logomarsino J.V. (2016). Bacterial Lipopolysaccharide, Lipopolysaccharide-Binding Protein, and Other Inflammatory Markers in Obesity and After Bariatric Surgery. Metab. Syndr. Relat. Disord..

[57] Papetti L., Del Chierico F., Frattale I., Toto F., Scanu M., Mortera S.L., Rapisarda F., Di Michele M., Monte G., Ursitti F. (2024). Pediatric migraine is characterized by traits of ecological and metabolic dysbiosis and inflammation. J. Headache Pain.

[58] Taheri S. (2017). Effect of exclusion of frequently consumed dietary triggers in a cohort of children with chronic primary headache. Nutr. Health.

[59] Hering-Hanit R., Gadoth N. (2003). Caffeine-induced headache in children and adolescents. Cephalalgia.

[60] Juliano L.M., Griffiths R.R. (2004). A critical review of caffeine withdrawal: Empirical validation of symptoms and signs, incidence, severity, and associated features. Psychopharmacology.

[61] Watemberg N., Matar M., Har-Gil M., Mahajnah M. (2014). The influence of excessive chewing gum use on headache frequency and severity among adolescents. Pediatr. Neurol..

[62] Arzani M., Jahromi S.R., Ghorbani Z., Vahabizad F., Martelletti P., Ghaemi A., Sacco S., Togha M., School of Advanced Studies of the European Headache Federation (EHF-SAS) (2020). Gut-brain Axis and migraine headache: A comprehensive review. J. Headache Pain.

[63] Gazerani P. (2020). Migraine and Diet. Nutrients.

[64] Hindiyeh N.A., Zhang N., Farrar M., Banerjee P., Lombard L., Aurora S.K. (2020). The Role of Diet and Nutrition in Migraine Triggers and Treatment: A Systematic Literature Review. Headache.

[65] Kotchetkoff E.C.D.A., Oliveira L.C.L.D., Sarni R.O.S. (2024). Elimination diet in food allergy: Friend or foe?. J. Pediatr..

[66] Wickman B.E., Enkhmaa B., Ridberg R., Romero E., Cadeiras M., Meyers F., Steinberg F. (2021). Dietary Management of Heart Failure: DASH Diet and Precision Nutrition Perspectives. Nutrients.

[67] Martínez-González M.A., Salas-Salvadó J., Estruch R., Corella D., Fitó M., Ros E. (2015). Benefits of the Mediterranean Diet: Insights From the PREDIMED Study. Prog. Cardiovasc. Dis..

[68] Sondhi V., Agarwala A., Pandey R.M., Chakrabarty B., Jauhari P., Lodha R., Toteja G.S., Sharma S., Paul V.K., Kossoff E. (2020). Efficacy of Ketogenic Diet, Modified Atkins Diet, and Low Glycemic Index Therapy Diet Among Children With Drug-Resistant Epilepsy: A Randomized Clinical Trial. JAMA Pediatr..

[69] Tereshko Y., Bello S.D., Di Lorenzo C., Pez S., Pittino A., Sartor R., Filippi F., Lettieri C., Belgrado E., Garbo R. (2023). 2:1 ketogenic diet and low-glycemic-index diet for the treatment of chronic and episodic migraine: A single-center real-life retrospective study. J. Headache Pain.

[70] Younas A., Naqvi S.A., Khan M.R., Shabbir M.A., Jatoi M.A., Anwar F., Inam-Ur-Raheem M., Saari N., Aadil R.M. (2020). Functional food and nutra-pharmaceutical perspectives of date (*Phoenix dactylifera* L.) fruit. J. Food Biochem..

[71] Gibson G.R., Hutkins R., Sanders M.E., Prescott S.L., Reimer R.A., Salminen S.J., Scott K., Stanton C., Swanson K.S., Cani P.D. (2017). Expert consensus document: The International Scientific Association for Probiotics and Prebiotics (ISAPP) consensus statement on the definition and scope of prebiotics. Nat. Rev. Gastroenterol. Hepatol..

[72] Holscher H.D. (2017). Dietary fiber and prebiotics and the gastrointestinal microbiota. Gut Microbes.

[73] Okburan G., Kızıler S. (2023). Human milk oligosaccharides as prebiotics. Pediatr. Neonatol..

[74] Ghavami A., Khorvash F., Heidari Z., Khalesi S., Askari G. (2021). Effect of synbiotic supplementation on migraine characteristics and inflammatory biomarkers in women with migraine: Results of a randomized controlled trial. Pharmacol. Res..

[75] Anhê F.F., Marette A. (2017). A microbial protein that alleviates metabolic syndrome. Nat. Med..

[76] Martami F., Togha M., Seifishahpar M., Ghorbani Z., Ansari H., Karimi T., Jahromi S.R. (2019). The effects of a multispecies probiotic supplement on inflammatory markers and episodic and chronic migraine characteristics: A randomized double-blind controlled trial. Cephalalgia.

[77] Reina F., Salemi G., Capizzi M., Lo Cascio S., Marino A., Santangelo G., Santangelo A., Mineri M., Brighina F., Raieli V. (2023). Orofacial Migraine and Other Idiopathic Non-Dental Facial Pain Syndromes: A Clinical Survey of a Social Orofacial Patient Group. Int. J. Environ. Res. Public Health.

